# 

**Published:** 1879-04

**Authors:** 


### BOOKS AND PAMPHLETS RECEIVED.

Health Primers; four vols. 1. Exercise and Training; by C. H. Ralfe, M.D. 2. Alcohol, its Uses and Abuses; by W. S. Greenfield, M.D. 3. The House and its Surroundings. 4. Premature Death, its Promotion and Prevention. D. Appleton & Co., New York.

Health and How to Promote It. By R. McSherry, M.D. Cl., pp. 185. 1879. Appleton & Co., New York.

General Surgical Pathology and Therapeutics, in 51 Lectures. By T. Billroth. Translated from German Ed. by C. E. Hackley, M.D. Cl., pp. 773. 1879.

Clinical Lectures on Diseases Peculiar to Women. By Lomb Atterill, M.D. Fifth Ed., revised and enlarged. Phil., Lindsay & Blakiston, 1879. pp. 342.

A Manual for the Practice of Surgery. By Thos. Bryant, F.R.C.S., etc. 2nd Amer., from 3d Revised Eng. Ed. Phil., H. C. Lea, 1879. Leather, pp 945.

Yellow Fever. By Thos. O. Summers, M.D.

Index to Original Communications in the Med. Jours. of U. S. and Canada for 1877. By W. D. Chapin. Cl., pp. 48.

Transactions of the Medical Society of the State of Tennessee at its Forty-fifth Annual Meeting. 1878.

First Annual Report of the State Board of Health of Illinois. 1878.

Transactions of the American Dermatological Association at the Second Meeting held at Saratoga Springs, Aug. 27, 28 and 29. 1879. Rep. from *New York Med. Jour.*, Oct., Dec., 1878, and Jan. and Feb., 1879.

Revue de la Clinique de Maladies des Femmes de la Faculté de Médecine de Lyon. By Joanni Rendu.

Points in Diagnosis of Hepatic Affections. By E. J. Janeway, M.D.

Circular in Reference to American Archæology. Smithsonian Miscellaneous Collection. 316.

In Memoriam, Dr. Landon R. Longworth. By F. Forchheimer, M.D.

Excerpta from the Annual Report to the Board of Health for 1878. By J Holt, M.D., Sanitary Inspector of the 4th District of New Orleans.

Information for the Taxpayers; Medical Laws; the State Board of Health; Finances.

Memorandum of the American Public Health Association on Legislation Affecting the Public Health.

Report on Aconitia in Trigeminal Neuralgia. By E. C. Seguin, M.D. Reprint from *New York Med. Jour.*, Dec., 1878.

Cases Illustrating Two Rare Diseases of the Eyelids—Syphilitic Gummata of the Conjunctiva. By Chas. Stedman Bull, M.D. Rep. from the Transactions of the Amer. Ophthalmological Society, 1878.

Diphtheria and Its Treatment. By Ch. H. S. Davis, M.D. Rep. from *Richmond and Louisville Med. Journal.*

Report of the Pennsylvania Hospital for the Insane for the Year. By Thos. S. Kirkbridge, M.D.

Report of Investigations into the Pathogeny of Diphtheria Conducted by Ed. Curtis, M.D., and Thos. E. Satterthwaite, M.D.

Address to the Medical Profession. By W. A. Hammond, M.D.

A Case of Inflammatory Fungoid Neoplasm. By Louis A. Duhring, M.D. Reprint from the *Archives of Dermatology* for Jan., 1879.

A Clinical Lecture on Anæsthetic Leprosy. By James Nevins Hyde, M.D. Reprint from *Amer. Practitioner*, Feb., 1879.

Medicine, the Present and Future. By J. W. Compton, M.D. Rep. from *St. Louis Med. and Surgical Journal*, Jan., 1878.

Proceedings of the Board of Experts Authorized by Congress to Investigate the Yellow Fever Epidemic of 1878.

Notes on Salicylic Acid. By Ed. R. Squibb, M.D. Read before the Medical Society of the State of New York, Feb. 2, 1875.

---

MEDICAL REMUNERATION.—Doctor: "Um! most insolent!" (To his wife) "Listen to this, my dear;" (reads letter aloud) "Sir: I enclose P. O. order for thirteen shillings and sixpence, hoping it will do you as little good as your two small bottles of 'physic' did me."—*Punch.*

### ANNOUNCEMENTS FOR THE MONTH.

### SOCIETY MEETINGS.

Chicago Medical Society—Mondays, April 7 and 21.

West Chicago Medical Society—Mondays, April 14 and 28.

### CLINICS.

MONDAY.

Eye and Ear Infirmary—2 p. m., Ophthalmological, by Prof. Holmes; 3 p. m., Otological, by Prof. Jones.

Mercy Hospital—1:30 p. m., Surgical, by Prof. Andrews.

Rush Medical College—2 p. m., Dermatological and Venereal, by Prof. Hyde; 3 p. m., Medical, by Dr. Bridge.

Woman's Medical College—2 p. m., Dermatological, by Dr. Maynard.

TUESDAY.

Cook County Hospital—2 to 4 p. m., Medical and Surgical Clinics.

Mercy Hospital—1:30 p. m., Medical, by Prof. Hollister.

WEDNESDAY.

Chicago Medical College—1:30 p. m., Eye and Ear, by Prof. Jones.

Rush Medical College—3:30 to 4:30 p. m., Diseases of the Chest, by Dr. E. Fletcher Ingals.

THURSDAY.

Chicago Medical College—1:30 p. m., Medical, by Prof. Quine.

Rush Medical College—3 p. m., Diseases of the Nervous System, by Prof. Lyman.

Eye and Ear Infirmary—2 p. m., Ophthalmological, by Dr. Hotz.

FRIDAY.

Cook County Hospital—2 to 4 p. m., Medical and Surgical Clinics.

Mercy Hospital—1:30 p. m., Medical, by Prof. Davis.

SATURDAY.

Rush Medical College—2 p. m., Surgical, by Prof. Gunn.

Chicago Medical College—2 p. m., Surgical, by Prof. Isham; 3 p. m., Neurological, by Prof. Jewell.

Woman's Medical College—11 a. m., Ophthalmological, by Dr. Montgomery.

Daily Clinics, from 2 to 4 p. m., at the Central Free Dispensary, and at the South Side Dispensary.

